# Time-course transcriptome and WGCNA reveal tissue-specific genes in ‘White BK-1’ rye during early cold acclimation stage

**DOI:** 10.3389/fpls.2026.1936346

**Published:** 2026-09-07

**Authors:** Dan Wang, Wei Ren, Xianguo Wang

**Affiliations:** China Agricultural University College of Grassland Science and Technology, Beijing, China

**Keywords:** early cold acclimation stage, rye, tissue-specific, transcriptome, WGCNA

## Abstract

**Introduction:**

The early stage of cold acclimation (CA) is a critical period during which plants gradually transition from cold sensing to the establishment of acquired freezing tolerance. Winter rye (*Secale cereale* L.) is a cereal crop with superior cold hardiness. However, the temporal transcriptional dynamics during early CA and the tissue-specific expression patterns between leaves and roots remain poorly understood.

**Methods:**

We evaluated the growth and freezing survival of ‘White BK-1’ during long-term CA and profiled leaf and root transcriptomes at multiple time points during the first 48 h of CA.

**Results:**

Following CA, enhanced freezing tolerance in rye was associated with growth inhibition. Time-course transcriptome analysis of leaves and roots during early CA (0-48 h) revealed distinct transcriptional reprogramming patterns. At 2 h, 2,585 and 137 DEGs (Differentially expressed genes) were identified in roots and leaves, respectively. DEG numbers peaked at 48 h in both tissues, reaching 8,795 in leaves and 8,984 in roots. The leaf responses were initially dominated by photosynthesis-related pathways, followed by the upregulation of protective metabolism, such as trehalose biosynthesis, glutathione metabolism, and phenylpropanoid biosynthesis. The response in roots was characterized by rapid transcriptional reprogramming, followed by plant hormone signal transduction and damage clearance mechanisms, including ABA/ethylene responses, autophagy, and linoleic acid metabolism, several genes associated with ABA signaling pathways, as well as a gene encoding the DELLA inhibitor RHT1 (*SECCE4Rv1G0219050*), were significantly upregulated. In total, 764 DETFs (Differentially Expressed Transcription Factors) were identified, mainly from the ERF, MYB, WRKY, bHLH, and NAC families. Moreover, *SECCE5Rv1G0340400* (*DREB1H*, ERF family), *SECCE5Rv1G0304800* (*CPRF2*, bZIP family), and *SECCE3Rv1G0171840* (*CDF2*, Dof family) were upregulated in both tissues. WGCNA (Weighted gene co-expression network analysis) identified modules closely associated with 48 h cold treatment in ‘White BK-1’ leaves and roots. Within the ICE-CBF-COR signaling pathway, *SECCE7Rv1G0464580* (*COR27*) and *SECCE5Rv1G0354870* (*COR413PM1*) exhibited the highest connectivity in the leaf and root subnetworks, respectively.

**Discussion:**

Our study provides insight into tissue-specific transcriptional responses in rye leaves and roots during early CA, identifying candidate genes and co-expression modules potentially relevant to cereal cold tolerance.

## 1 Introduction

Cold is a major abiotic stress that limits plant geographical distribution and productivity worldwide. According to temperature range, it is generally divided into chilling (0-15 °C) and freezing (< 0 °C) (Hwarari et al., 2022; Ding et al., 2024; Zeng et al., 2026). When exposed to low but non-freezing temperatures, overwintering plants such as winter wheat and winter rye undergo cold acclimation (CA), acquiring freezing tolerance (Thomashow, 1999; Shi et al., 2018; Sasaki and Imai, 2023). This process involves the accumulation of protective compounds like proline, soluble sugars, antioxidants, antifreeze proteins, late embryogenesis abundant proteins and cold shock proteins, as well as changes in metabolism, photosynthesis and membrane composition, all of which enhance subsequent freezing tolerance (Thomashow, 2010; Rapacz et al., 2014; Seydel et al., 2022; Yu et al., 2023). The early phase of CA represents the transition from low temperature perception to the establishment of acquired freezing tolerance. This period, usually spanning the first several hours to days after exposure to low temperature, involves rapid transcriptional changes linking temperature sensing with downstream protective responses (Luklová et al., 2025). In Arabidopsis, cold-responsive transcripts have been classified into three temporal groups: early transiently induced genes (0–12 h), intermediate genes (12 h-48 h), and late acclimation genes (> 48 h) (Hannah et al., 2005). Cold exposure triggers extensive transcriptional changes within 30 min to 1 h, and approximately 86% of cold-responsive genes differ between roots and shoots, indicating substantial organ-specific transcriptional responses to cold (Kreps et al., 2002; Kilian et al., 2007). Compared with shoots, roots experience distinct soil temperature and water conditions and are particularly vulnerable to freezing-induced dehydration. Previous studies indicate that root CA involves the coordinated regulation of cellular protection, water uptake, and root growth (Khanna and Daggard, 2006; Peppino Margutti et al., 2018; Ambroise et al., 2020). In overwintering cereals, defining these root-specific programs is essential because winter survival depends not only on freezing tolerance itself, but also on the capacity of roots to maintain function and support regrowth after cold stress (Ambroise et al., 2020; Karlova et al., 2021). However, cold responses in roots remain less understood than those in shoots, especially during the early stages of CA in overwintering cereals.

In *Triticeae*, the perception of low temperature at the cell surface initiates CA and rapidly triggers Ca^2+^ signaling and transcriptional reprogramming. These early responses coordinate carbohydrate accumulation, redox protection, growth restraint, and other adaptive processes required for freezing tolerance (Liu et al., 2022; Kosová et al., 2025). The CBF-*COR* pathway is a central component of this response, activating the expression of numerous protective genes, including those encoding COR proteins and LEA/dehydrin proteins, such as WCS120 and WCOR14. These proteins accumulate during CA and help cells withstand the dehydration caused by extracellular ice formation (Ganeshan et al., 2008; Kosová et al., 2011; Hwarari et al., 2022). In winter wheat, increased TaICE1 stability is also associated with accelerated starch remobilization and soluble sugar accumulation, which enhance osmotic protection and help maintain antioxidant capacity under low-temperature stress (Peng et al., 2021). Additionally, phytohormones also regulate cold responses through both CBF-dependent and CBF-independent pathways (Shi et al., 2015; Kaya et al., 2024). In winter wheat, CA markedly alters the homeostasis of Cytokinins (CKs), Gibberellins (GAs), Auxin (IAA), Jasmonic acid (JA), Salicylic acid (SA) and Abscisic acid (ABA) in leaves and roots (Wang et al., 2023). In the ABA signaling pathway, cold stress induces *TaSnRK2.3*, *TaSnRK2.4*, and *TaSnRK2.8*. Heterologous expression of *TaSnRK2.3* or *TaSnRK2.8* enhances cold tolerance, accompanied by increased *COR* expression and proline accumulation (Zhang et al., 2010). JA accumulation and methyl JA treatment are likewise associated with higher *COR* expression and increased SOD and POD activities (Talanova et al., 2018; Repkina et al., 2021). The interactions between CBF family proteins and GA signaling link CA to growth inhibition. *CBF* overexpression reduces bioactive GA levels and leads to dwarfing and delayed flowering (Soltész et al., 2013; Lantzouni et al., 2020). Plant hormones contribute differently to low-temperature responses, and the expression profiles of their signaling genes may help characterize hormone-related responses to cold stress.

Rye (*Secale cereale* L.) is a strongly cold-hardy cereal that maintains photosynthetic activity, membrane integrity, and meristem viability during prolonged sub-zero exposure, enabling stable cultivation along the northern margins of European and Eurasian agriculture (Behre, 1992; Hackauf et al., 2022). Classical genetic studies have shown that major determinants of rye freezing tolerance reside on chromosome 5R, a region syntenic with the wheat *Fr-A1/Fr-A2* and barley *Fr-H1/Fr-H2* gene complexes (Plaschke et al., 1993; Erath et al., 2017). The *ScCbf* cluster on 5R contains multiple members of the *CBF-III* and *CBF-IV* subfamilies, and transcript accumulation of several *ScCbf-IVd* and *ScCbf-IIIc* paralogues under low temperature is associated with field-derived LT50 values across rye germplasm (Campoli et al., 2009; Li et al., 2011; Rabanus-Wallace et al., 2021). Because rye is closely related to wheat and barley, it serves as a genetic resource for studying the cold tolerance of overwintering *Triticeae* crops (Bahrani et al., 2021; Båga et al., 2022; Sun et al., 2022). Rye has also served as a donor of cold-tolerance alleles in wheat improvement, notably through 1BL/1RS chromosome translocations and the development of synthetic *triticale* (Plaschke et al., 1993; Sun et al., 2022; Han et al., 2025). Despite its agronomic and genetic importance, studies of rye responses to cold stress remain limited, and omics-based analyses have largely focused on aerial tissues. In et al. used suppression subtractive hybridization and cDNA library screening in rye leaves recovered after CA and identified peptide methionine sulfoxide reductase (PMSR), a protein implicated in the repair of oxidatively damaged proteins under low temperature (In et al., 2005). Using the ‘Weining’ rye genome as a reference, Li et al. performed RNA-Seq on young leaves of two rye genotypes exposed to 4 °C and reported genotype-dependent transcriptional responses to cold treatment (Li et al., 2025). However, compared with wheat and barley, rye remains underrepresented in cold-response genomics, such aspects as its root transcriptome, temporal features of early CA, and tissue-specific expression patterns between leaves and roots remain poorly characterized.

This study found that CA improved rye survival following exposure to extreme freezing stress while reducing plant height and aboveground biomass accumulation. Although our previous work explored the physiological mechanisms of this line during CA (Yang et al., 2024a, Yang et al., 2024b), yet the transcriptional basis for these adaptations remains unclear. Therefore, using the ‘Lo7’ rye genome as the reference, we analyzed the leaf and root transcriptomes of ‘White BK-1’ during the first 48 h of CA. The time-course analysis revealed pronounced tissue specificity in the early CA transcriptome, rye roots underwent transcriptional reprogramming, mainly involving hormone signaling, metabolism, antioxidant defense, circadian regulation, and DREB/CBF-related networks. Leaf responses were more closely associated with photosynthesis, energy metabolism, and stress-protective pathways. In addition, several candidate genes associated with early CA have been identified, including *SECCE5Rv1G0340400* (*DREB1H*), *SECCE5Rv1G0304800* (*CPRF2*), *SECCE3Rv1G0171840* (*CDF2*), *SECCE7Rv1G0464580* (*COR27*), *SECCE5Rv1G0354870* (*COR413PM1*), and *SECCE4Rv1G0219050* (*RHT1*), etc. These results extend the current genomic resources for rye cold responsive studies and provide a transcriptional framework for the future analysis of frost resistance in winter rye and related *Triticeae* crops.

## 2 Materials and methods

### 2.1 Plant materials, growth condition, and freezing tolerance treatment

The rye accession ‘White BK-1’, a Russian winter rye with high winter hardiness, was used in this study (Yang et al., 2024a, Yang et al., 2024b). Seeds were surface-sterilized in 3% (v/v) NaClO for 5 min, rinsed five times with sterile distilled water, and germinated in 10 cm × 10 cm trays containing washed quartz sand. Seedlings were grown in a controlled-environment chamber at 20 °C/16 °C day/night temperature, with a 16 h/8 h light/dark photoperiod and a photosynthetic photon flux density (PPFD) of 300 μmol m^−2^ s^−1^. After 7 d, uniform seedlings were selected, the roots were gently washed free of residual sand, and the seedlings were transplanted into 5 cm × 5 cm trays filled with substrate soil, with 10 seedlings of the same accession per tray. After 3 d of establishment, initial plant height was recorded as H0, and shoot biomass was determined from parallel seedlings grown under identical conditions and recorded as W0. Plants were then divided into non-acclimated (NA) and cold-acclimated (CA) treatments, with four biological replicates per accession and treatment. NA and CA treatments were conducted according to Yu et al. with minor modifications. NA plants were maintained at 20 °C/16 °C day/night temperature, 16 h/8 h light/dark photoperiod, and 300 μmol m^−2^ s^−1^ PPFD for 2 weeks. CA plants were grown at 5 °C/2 °C day/night temperature, 8 h/16 h light/dark photoperiod, and 300 μmol m^−2^ s^−1^ PPFD for 7 weeks (Yu et al., 2001). The longer duration of the CA treatment was selected based on previous studies, as plant growth and development are markedly slower under low-temperature conditions. Plants grown for 3 weeks at 20 °C/16 °C and those exposed to 5 °C/2 °C for 7 weeks have been shown to reach comparable developmental stages (Krol et al., 1984; Griffith and McLntyre, 1993). At the end of each treatment, plant height was recorded as H1 or H2, respectively, and shoot biomass was determined from parallel plants under the corresponding treatment and recorded as W1 or W2.


$$\text{NA: The relative height growth rate}\left(\text{RHGR}\right)=\frac{lnH1-lnH0}{14}\text{ cm/cm}\cdot \text{d}$$

$$\text{CA: RHGR}=\frac{lnH2-lnH0}{49}\text{ cm/cm}\cdot \text{d}$$

$$\text{NA: The relative biomass growth rate}\left(\text{RBGR}\right)=\frac{lnW1-lnW0}{14}\text{ g/g}\cdot \text{d}$$

$$\text{CA: RBGR}=\frac{lnW2-lnW0}{49}\text{ g/g}\cdot \text{d}$$

Plants retained for freezing assessment were then exposed to -20 °C for 3 h and subsequently transferred back to the normal growth conditions for 7 d of recovery. Survival was assessed by counting the number of regrown plants in each tray, and survival rate was expressed as the percentage of surviving plants relative to the number of plants before freezing. This study aimed to evaluate the phenotypic effects of CA on freezing tolerance in ‘White BK-1’.


$$\text{Survival rate}=\frac{Survival\;number}{Total\;number}\times 100\%$$

### 2.2 RNA sequencing and differential expression analysis

RNA-Seq was performed to characterize early transcriptional responses during CA. Ten-day-old seedlings were transferred to 2 °C under the same photoperiod and light intensity used for normal growth, so as to avoid confounding early cold-response transcriptional changes with effects caused by an altered photoperiod. Leaves and roots were sampled at 0, 2, 12, 24, and 48 h after the start of treatment; the 0 h samples were collected immediately before transfer to 2 °C. For each tissue and time point, three independent biological replicates were collected. Samples were wrapped in aluminum foil, immediately frozen in liquid nitrogen, and stored at -80 °C until RNA extraction.

Total RNA was extracted with TRIzol^®^ reagent following the manufacturer’s protocol. RNA concentration and purity were measured on a NanoDrop 2000 spectrophotometer, while RNA integrity was evaluated by agarose gel electrophoresis and Agilent 2100 Bioanalyzer analysis. Strand-specific mRNA libraries were generated with the Illumina^®^ Stranded mRNA Prep, Ligation kit. Libraries with an insert size of approximately 300 bp were selected, subjected to 15 cycles of PCR amplification with Phusion High-Fidelity DNA Polymerase, and quantified using a Qubit 4.0 fluorometer. Paired-end sequencing was carried out on an Illumina NovaSeq X Plus platform to generate 150-bp reads.

Adapters, low-quality reads, and short reads were removed from the raw data with fastp (Version 0.23.4) (Chen et al., 2018). Clean reads were aligned to the *Secale cereale* ‘Lo7’ reference genome using HISAT2 (Version 2.2.1) (Kim et al., 2015). The reference genome assembly (GCA_902687465.1; Rye_Lo7_2018_v1p1p1; BioProject: PRJEB35356) and the corresponding gene annotation files were downloaded from Ensembl Plants (https://plants.ensembl.org/Secale_cereale/Info/Index) (Rabanus-Wallace et al., 2021). StringTie (Version 2.2.1) was used for reference-guided transcript assembly and expression quantification (Pertea et al., 2015). Genes with a TPM (transcripts per million) > 0 in at least one sample were considered to have detectable expression. Novel transcripts were identified by comparing the StringTie-assembled transcripts with the reference annotation using gffcompare (). Transcripts assigned the class code “u” were considered novel intergenic transcripts, and the corresponding gene loci were defined as novel genes. Differential expression analysis was performed in DESeq2 (Version 1.42.0) using gene-level counts, with TPM values were used to represent normalized expression abundance (Love et al., 2014). Genes with FDR (false discovery rate)< 0.05 and |log_2_ fold change| ≥ 2 were considered differentially expressed.

### 2.3 GO and KEGG enrichment analysis of DEGs

GO annotations were taken from the ‘Lo7’ rye genome annotation and grouped into biological process (BP), cellular component (CC), and molecular function (MF), while KEGG Orthology and pathway annotations were derived from the KEGG database. Enrichment analysis was performed with Goatools (Version 1.4.4) for GO terms and with SciPy for KEGG pathways (Klopfenstein et al., 2018). Fisher’s exact test was used in both analyses, and terms or pathways with a Benjamini-Hochberg-adjusted *P* value< 0.05 were considered significantly enriched. Expressed genes with the corresponding GO or KEGG annotations were used as the background.

### 2.4 Weighted gene co-expression network analysis

Co-expression networks were built using the WGCNA package in R v4.2.2 (Version 1.63) (Langfelder and Horvath, 2008). Genes with both mean TPM< 2 and coefficient of variation< 0.1 were excluded from the normalized expression matrix before network construction. Pairwise adjacency matrices were then calculated using the remaining genes. To select the soft-thresholding power, candidate powers ranging from 1 to 20 were evaluated according to the scale-free topology criterion. A power of 9 was selected for the leaf network, whereas a power of 18 was selected for the root network. These powers provided acceptable scale-free topology fits, with R^2^ values of 0.6366 and 0.8543, respectively, while maintaining adequate mean connectivity (**Supplementary Figure 2**). The resulting adjacency matrices were converted into topological overlap matrices (TOMs), and hierarchical clustering was performed based on TOM dissimilarity. Module detection used minModuleSize = 30, minKMEtoStay = 0.30, and mergeCutHeight = 0.25. Following module detection and merging, 11 modules were identified in leaves and 25 modules were identified in roots. Module eigengenes were correlated with CA duration separately in leaves and roots. Modules were considered to be associated with CA duration when the module-trait correlation met the predefined criteria of |r| ≥ 0.6 and a Benjamini-Hochberg adjusted *P*<0.05. For each treatment-associated module, module membership (MM) was calculated as the correlation between each gene and the corresponding module eigengene, whereas gene significance (GS) was defined as the association between gene expression and the 0 h versus 48 h CA contrast (Azam et al., 2023). DEGs with MM ≥ 0.8 and |GS| ≥ 0.6 were retained as module-associated candidate genes. To further prioritize highly connected candidates, intramodular co-expression subnetworks were constructed from these selected genes based on topological overlap, edges with a TOM-based weight > 0.02 were retained.

### 2.5 Identification of transcription factors, differentially expressed TFs, ICE-CBF-*COR* genes, and hormone genes

Putative TFs were identified from the “White BK-1’ protein set using hmmscan against PlantTFDB HMM profiles (version 5.0; http://planttfdb.gao-lab.org/) (Jin et al., 2017a) and BLASTP against the PlantTFDB TF dataset, using an E-value threshold of 1 × 10^−5^. Family assignments were based on PlantTFDB annotations and the corresponding DNA-binding domains. TF genes that overlapped with the DEGs were considered DETFs. *Arabidopsis* ICE, CBF/DREB1 and COR proteins were used as queries to search the ‘White BK-1’ protein database with BLAST in TBtools (Chen et al., 2023), with an E-value cutoff of 1 × 10^−5^. Candidate hits were further screened based on GO annotations, NCBI BLASTP (https://blast.ncbi.nlm.nih.gov/Blast.cgi) and annotation consistency with homologous genes from *Triticeae* species. Separate WGCNA networks were built for leaves and roots. TOM-based weights among the selected genes were extracted from each network, and connections with weights > 0.02 were visualized in Cytoscape software (Shannon et al., 2003). Hormone signaling genes were selected from KEGG Orthology annotations corresponding to the Plant hormone signal transduction pathway (map04075).

### 2.6 qRT-PCR validation

Twelve genes were selected for RT-qPCR validation from the CA-responsive candidate list, including genes identified through WGCNA, TFs analysis, and differential expression analysis. The selection prioritized genes with significant differential expression, high module membership or intramodular connectivity, and representative candidates from the major regulatory groups observed in the study. Gene-specific primers were designed using Premier 5 and are listed in **Supplementary Table 10**. Total RNA was extracted from leaf and root samples collected at 0, 2, 12, 24, and 48 h of CA using the Eastep Super Total RNA Extraction Kit. First-strand cDNA was synthesized with UnionScript First-strand cDNA Synthesis Mix for qPCR with dsDNase, and RT-qPCR was carried out using GS AntiQ qPCR SYBR Green Fast Mix on a qTOWER3/G real-time PCR system. *ScActin* was used as the internal reference gene (Jung and Seo, 2019; Ding et al., 2021; Wang et al., 2026). Relative transcript levels were calculated using the 2^−ΔΔCt^ method, with the 0 h sample as the calibrator. Three biological replicates were analyzed for each sample, and graphs were generated using GraphPad Prism 9.

### 2.7 Statistical analysis

Data were organized in Microsoft Excel and analyzed using IBM SPSS and GraphPad Prism. Student’s t-test was used to compare the survival rates of ‘White BK-1’ after -20 °C freezing treatment, as well as the RHGR and RBGR between CA and NA plants within each genotype. Figures were prepared with GraphPad Prism, Adobe Photoshop, and Adobe Illustrator. Data are shown as the mean ± standard deviation. Asterisks indicate significant differences: * *P* < 0.05, ** *P* < 0.01, and *** *P* < 0.001.

## 3 Results

### 3.1 Phenotypic effects of CA on freezing tolerance in rye

**Figure 1:**
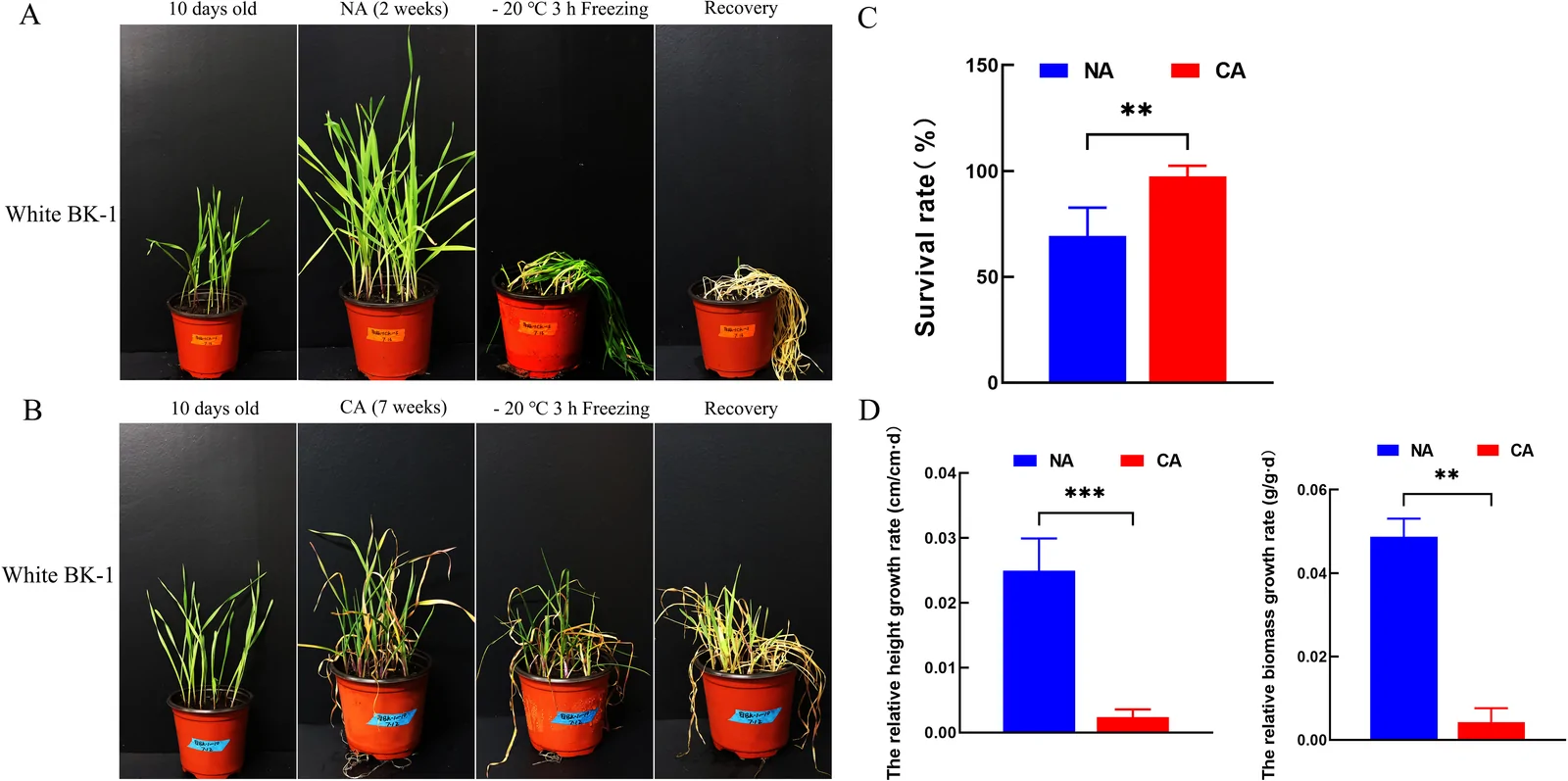
Effects of CA on freezing survival and growth in ‘White BK-1’. **(A)** Freezing phenotypes under NA conditions; **(B)** Freezing phenotypes under CA conditions; **(C)** Survival rates after post-freezing recovery under NA and CA; **(D)** Relative growth rates of rye height and shoot biomass under NA and CA. Significant differences were analyzed using the t-test (*P < 0.05; **P< 0.01; ***P < 0.001).

To evaluate the phenotypic effects of CA on freezing tolerance in rye, we compared post-freezing survival between NA and CA seedlings of ‘White BK-1’. Survival under NA conditions was 69.25%, whereas CA increased survival to 97.5% (**Figures 1A–C**). In parallel, RHGR and RBGR were significantly lower during CA than under NA conditions (**Figure 1D**). These results indicate that CA enhanced post-freezing survival in rye seedlings but was accompanied by reduced plant height and shoot biomass growth.

### 3.2 Analysis of raw RNA sequencing data

To investigate the dynamic transcriptional changes in ‘White BK-1’ rye during early CA, we performed RNA-seq on leaves and roots collected at 0, 2, 12, 24, and 48 h after cold treatment, with three biological replicates per tissue per time point. A total of 187.72 Gb of clean data was obtained from 30 libraries, with each sample yielding more than 5.46 Gb of clean reads. The Q20 and Q30 values exceeded 99.06% and 95.26%, respectively, and GC content ranged from 51.97% to 55.90%. Mapping rates to the reference ‘Lo7’ genome varied between 64.22% and 86.94%. Together, these results confirm the high quality of the sequencing data for subsequent analyses (**Supplementary Table 1**).

### 3.3 Analysis of differentially expressed genes during CA

**Figure 2:**
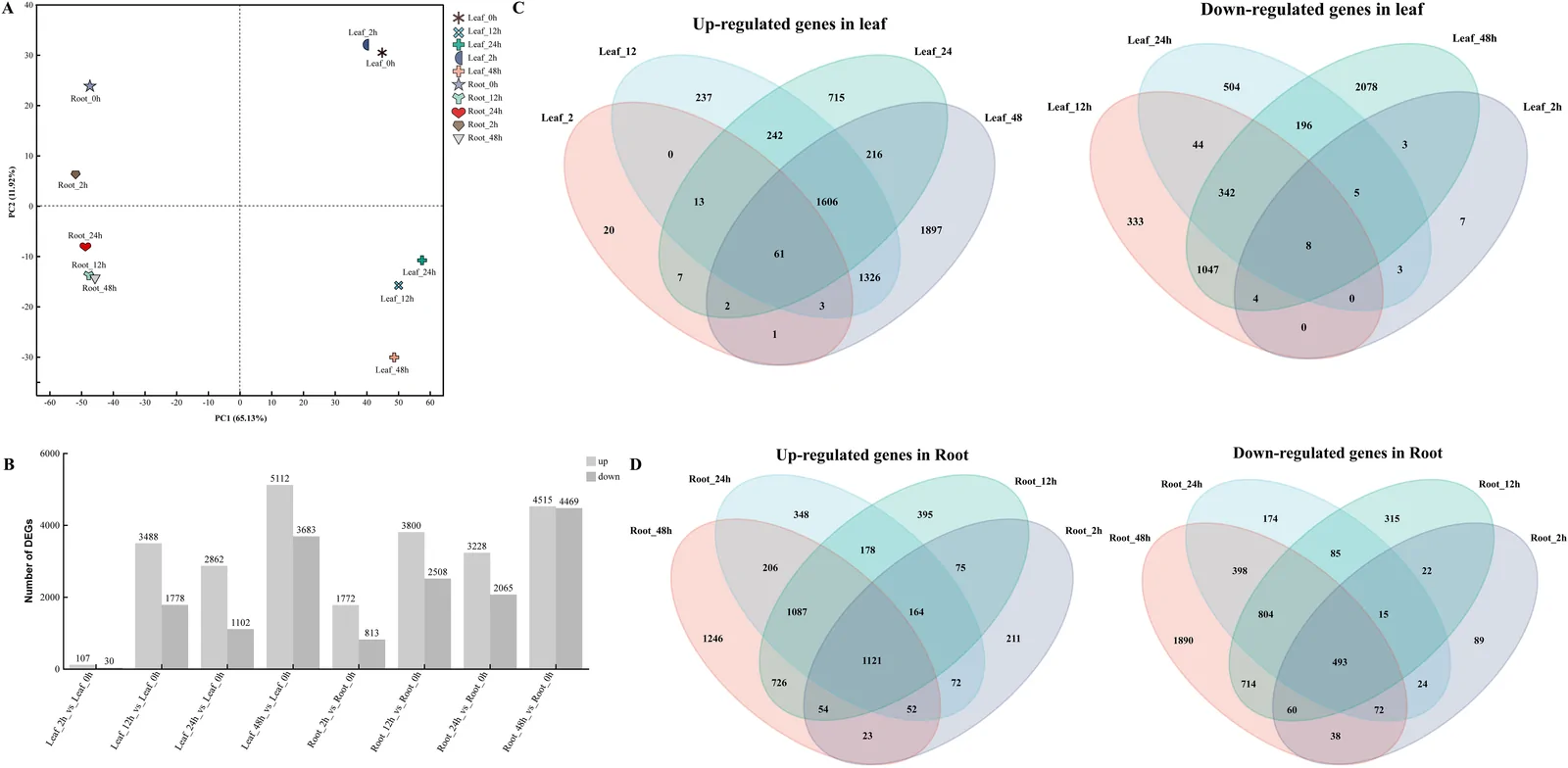
Transcriptomic profiling of ‘White BK-1’ rye leaves and roots during early CA. **(A)** Principal component analysis (PCA) of RNA-Seq data from leaf and root samples; **(B)** Numbers of up- and down-regulated differentially expressed genes (DEGs) in leaves and roots at each time point during early CA, using 0 h as the control; **(C)** Venn diagrams showing the overlap of up- and down-regulated DEGs in leaves across different CA time points; **(D)** Venn diagrams showing the overlap of up- and down-regulated DEGs in roots across different CA time points.

A total of 49,786 genes showed detectable expression, including 30,784 known genes and 19,002 novel genes. Principal component analysis (PCA) revealed clear transcriptomic separation between leaves and roots during early CA. PC1 and PC2 explained 65.13% and 11.92% of the total variance, respectively. Within each tissue, cold treated samples were clearly separated from controls, suggesting that early CA triggered significant transcriptome reprogramming in both leaves and roots. Notably, leaf samples at 2 h were closer to samples at 0 h, indicating that the response of the leaves within 2 h was mild. In contrast, root samples at 2 h were clearly separated from the control, while the 12, 24, and 48 h treatments formed a tight cluster (**Figure 2A**). Across the entire time course, 10,903 DEGs were identified in leaves and 11,140 in roots. At 2 h, the number of DEGs was relatively low in leaves, with 107 up-regulated and 30 down-regulated genes, whereas roots already displayed a much stronger response, with 1,772 up-regulated and 813 down-regulated genes. The largest number of DEGs was detected at 48 h, with 5,112 up-regulated and 3,683 down-regulated genes in leaves, and 4,515 up-regulated and 4,469 down-regulated genes in roots (**Figure 2B**). In addition, 61 genes were up-regulated and 8 genes were down-regulated in leaves across all four time points, compared with 1,121 up-regulated and 493 down-regulated genes in roots (**Figures 2C, D**). Together, these results indicate that during early CA in rye, leaves and roots show distinct transcriptional reprogramming pattern, roots respond faster than leaves, with pronounced transcriptional changes occurring as early as 2 h.

### 3.4 Temporal dynamics of GO enrichment among DEGs in ‘White BK-1’ leaves and roots during the early CA stage

**Figure 3:**
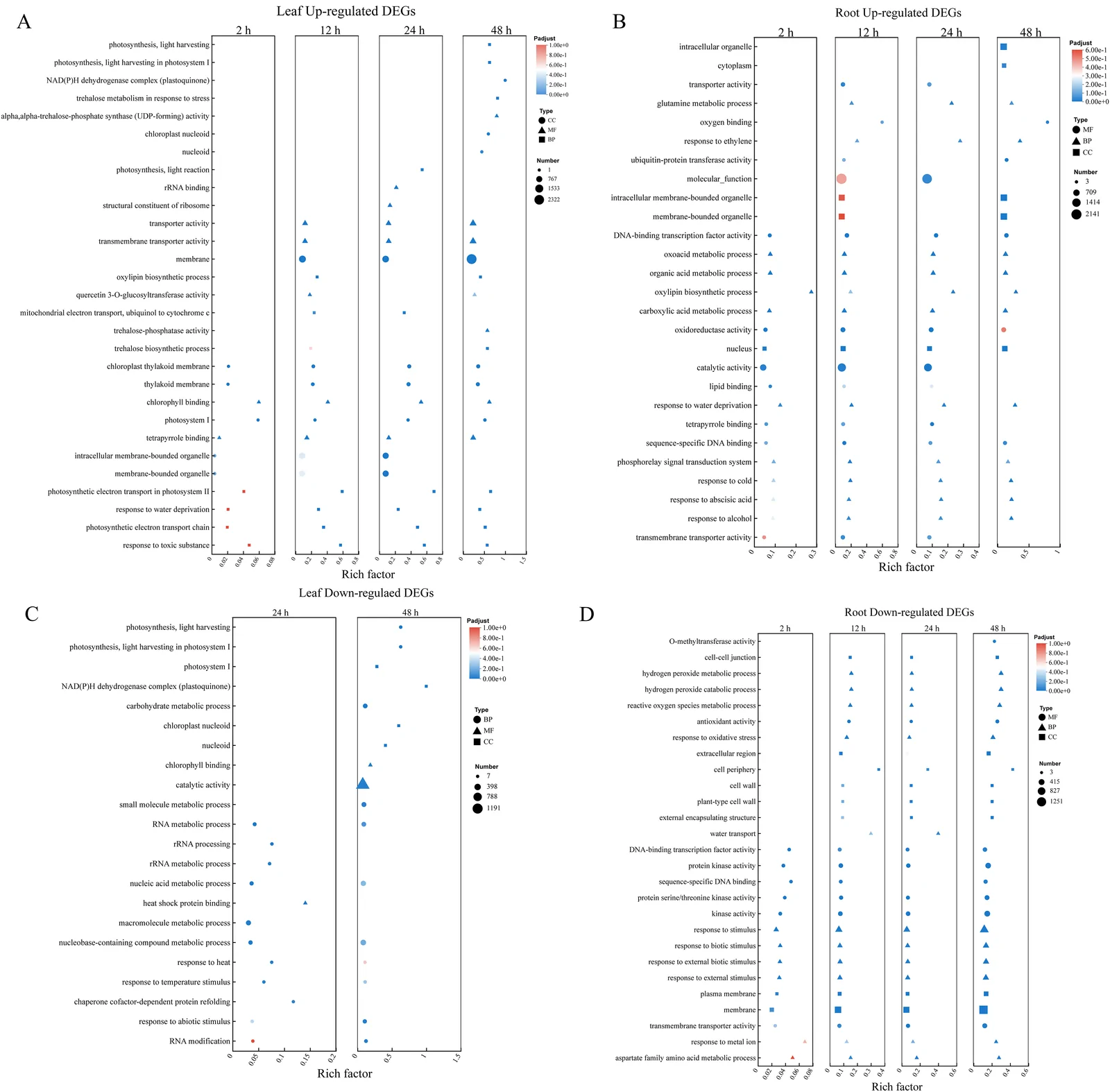
Gene ontology (GO) enrichment analysis of DEGs in leaves and roots of ‘White BK-1’ during early CA. **(A)** Up-regulated DEGs in leaves; **(B)** Up-regulated DEGs in roots; **(C)** Down-regulated DEGs in leaves; **(D)** Down-regulated DEGs in roots.

To characterize the functional features of DEGs in rye leaves and roots during early CA, GO enrichment analysis was performed. In leaves, up-regulated DEGs at 2 h were significantly enriched in terms related to photosynthetic structures and functions, including photosystem I (GO:0009522), chloroplast thylakoid membrane (GO:0009535), and tetrapyrrole binding (GO:0046906). During the 12–24 h period, terms such as photosynthetic electron transport in photosystem II (GO:0009772), response to toxic substance (GO:0009636), and photosynthesis, light reaction (GO:0019684) were further significantly enriched. At 48 h, trehalose-related terms, including trehalose metabolic process in response to stress (GO:0070413), alpha, alpha-trehalose-phosphate synthase (UDP-forming) activity (GO:0003825), and trehalose-phosphatase activity (GO:0004805), were also significantly enriched (**Figure 3A**). The down-regulated DEGs in leaves were mainly enriched at 24 and 48 h in terms associated with protein folding (GO:0006457), RNA processing (GO:0006396), photosynthetic light harvesting (GO:0009765), and catalytic activity (GO:0003824) (**Figure 3C**). These results suggest that rye leaves response to early CA involve first enriching genes related to processes associated with of photosynthesis, and then upregulating transcripts related to protective metabolic processes. In roots, the GO enrichment pattern differed markedly from that in leaves. At 2 h, up-regulated DEGs were significantly enriched in nucleus (GO:0005634), DNA-binding transcription factor activity (GO:0003700), and oxylipin biosynthetic process (GO:0031408), indicating rapid activation of transcriptional regulation and signaling after cold perception. During the 12–24 h period, terms such as response to cold (GO:0009409), glutamine metabolic process (GO:0006541), response to water deprivation (GO:0009414), and response to abscisic acid (GO:0009737) were significantly enriched. At 48 h, terms including response to ethylene (GO:0009723) and ubiquitin-protein transferase activity (GO:0004842) were also enriched (**Figure 3B**). The down-regulated DEGs in roots were enriched in terms related to the plasma membrane (GO:0005886), response to biotic stimulus (GO:0009607), kinase activity (GO:0016301), hydrogen peroxide metabolism (GO:0042743), reactive oxygen species metabolism (GO:0072593), antioxidant activity (GO:0016209), and cell wall-associated processes (GO:0005618, GO:0009505) (**Figure 3D**). Notably, the roots showed a significant transcriptional downregulation as early as 2 h after cold treatment, whereas a significant transcriptional downregulation in the leaves occurred at a later time point. This observation suggests that roots and leaves exhibit distinct transcriptional responses during early CA.

### 3.5 Temporal dynamics of KEGG pathway enrichment among DEGs in ‘White BK-1’ leaves and roots during the early CA stage

**Figure 4:**
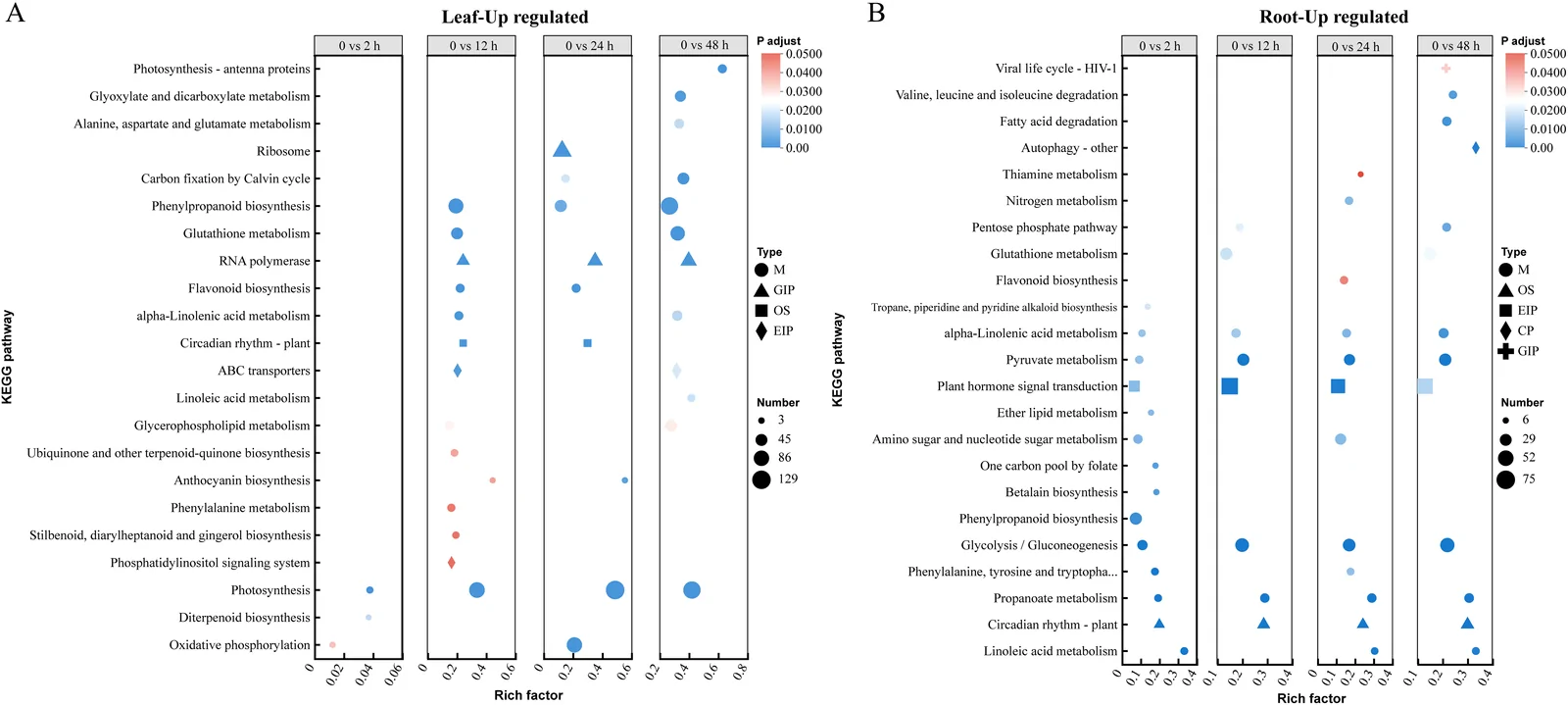
KEGG pathway enrichment analysis of up-regulated genes in leaves and roots during CA. **(A)** Up-regulated DEGs in leaves; **(B)** Up-regulated DEGs in roots.

To further characterize the metabolic pathways associated with early CA in rye, KEGG enrichment analysis was performed on the up- and down- regulated DEGs in leaves and roots, respectively (**Figure 4**; **Supplementary Figure 1**). In leaves, up-regulated DEGs at 2 h were significantly enriched in Photosynthesis (map00195), Oxidative phosphorylation (map00190), and Diterpenoid biosynthesis (map00904). At 12 h, the number of significantly enriched pathways increased from 3 to 15, including Anthocyanin biosynthesis (map00942), Linoleic acid metabolism (map00591), Phenylpropanoid biosynthesis (map00940), and Glutathione metabolism (map00480), etc. At 24 h, the number of genes enriched in Photosynthesis (map00195) increased substantially, and pathways such as RNA polymerase (map03020) and Ribosome (map03010) were also significantly enriched. At 48 h, Photosynthesis-antenna proteins (map00196), Photosynthesis (map00195), and Linoleic acid metabolism (map00591) remained significantly enriched (**Figure 4A**). In roots, 17 pathways were significantly enriched among up-regulated DEGs at 2 h, including Linoleic acid metabolism (map00591), Circadian rhythm-plant (map04712), Propanoate metabolism (map00640), Phenylpropanoid biosynthesis (map00940), and Glycolysis/Gluconeogenesis (map00010). At 12 h and 24 h, the number of enriched pathways was reduced to 8 and 12, respectively. Notably, Plant hormone signal transduction (map04075) was enriched throughout both phases. In addition, Glutathione metabolism (map00480), alpha-Linolenic acid metabolism (map00592), Pyruvate metabolism (map00620), and Glycolysis/Gluconeogenesis (map00010) pathways were also enriched at 12 h. At 24 h, significantly enriched pathways also included Amino sugar and nucleotide sugar metabolism (map00520), Flavonoid biosynthesis (map00941), Nitrogen metabolism (map00910), and Thiamine metabolism (map00730). At 48 h, 11 pathways remained significantly enriched, including Autophagy-other (map04136), Linoleic acid metabolism (map00591), Propanoate metabolism (map00640), Circadian rhythm-plant (map04712), and Valine, leucine and isoleucine degradation (map00280) (**Figure 4B**). These results indicate that during the early stage of CA, rye leaves and roots underwent significant metabolic pathway reprogramming, accompanied by both common and tissue-specific metabolic responses.

### 3.6 Prediction and expression pattern analysis of DETFs

**Figure 5:**
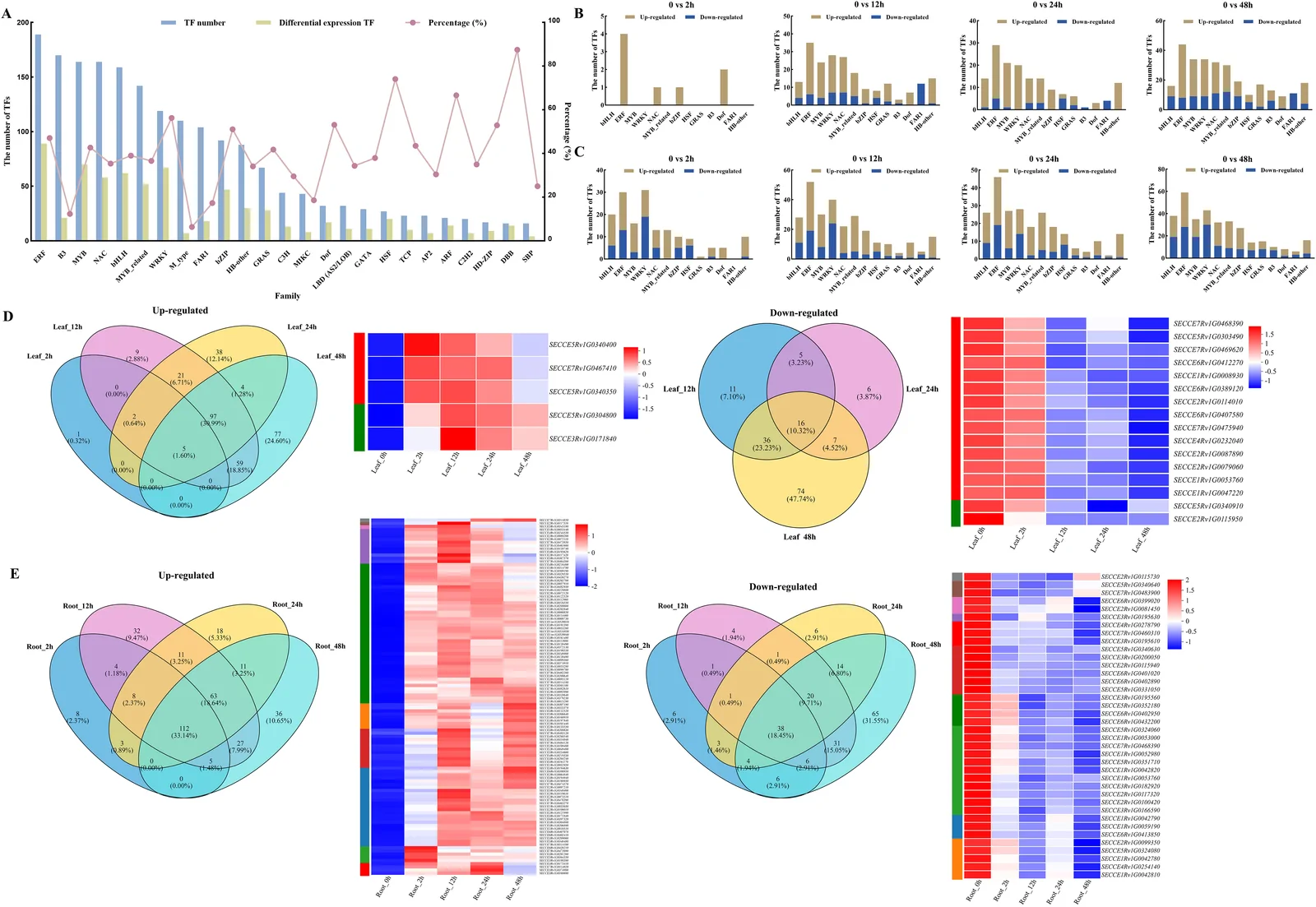
Identification and expression dynamics of differentially expressed transcription factors (DETFs) in ‘White BK-1’ leaves and roots during early CA. **(A)** Total TFs, DETFs, and DETF proportions in each TF family; **(B)** Up- and down-regulated DETFs in leaves at each time point relative to 0 h; **(C)** Up- and down-regulated DETFs in roots at each time point relative to 0 h; **(D)** Venn diagrams showing the overlap of up- and down-regulated DETFs in leaves across CA time points, with expression heatmaps of common DETFs; **(E)** Overlap of up- and down-regulated DETFs across time points in roots, with expression heatmaps of common DETFs.

To characterize TF mediated regulatory responses during early CA, TFs were identified from the transcriptome data of ‘White BK-1’. Based on conserved DNA-binding domains and sequence features, we identified 2063 transcription factors (TFs) across 47 families. Among the 25 largest families, ERF was the largest (189 members), followed by B3 (170), MYB (164), NAC (164), and bHLH (159), while SBP was the smallest (16). Of these TFs, 764 were differentially expressed and belonged to 42 families, with ERF containing the most DETFs (89), followed by MYB (70), WRKY (67), bHLH (62), and NAC (58) (**Supplementary Table 2**). When expressed as the proportion of differentially expressed members within each family, WRKY showed the highest proportion (56.30%), followed by ERF (47.09%), MYB (42.68%), bHLH (38.99%), and NAC (35.37%) (**Figure 5A**). DETFs exhibited clear tissue-specific temporal patterns during early CA. In leaves, the DETF numbers were low at 2 h but increased markedly from 12 h onward, reaching a maximum at 48 h (**Figure 5B**). In contrast, substantial numbers of both up-regulated and down-regulated TFs detected in roots as early as 2 h, and this was present at 12, 24, and 48 h (**Figure 5C**). The ERF family was consistently the predominant group among up-regulated DETFs in both tissues, whereas the down-regulated DETFs showed greater tissue specificity, with FAR1 being more prominent in leaves and WRKY in roots (**Figures 5B, C**). Venn diagram analysis showed that only 5 DETFs were up-regulated in leaves at all four time points (**Figure 5D**), whereas 112 up-regulated and 38 down-regulated DETFs were shared across all four time points in roots (**Figure 5E**). In addition, *SECCE5Rv1G0340400* (*DREB1H*, ERF family), *SECCE5Rv1G0304800* (*CPRF2*, bZIP family), and *SECCE3Rv1G0171840* (*CDF2*, Dof family) were up-regulated in both leaves and roots at all four time points, whereas two HSF family genes, *SECCE1Rv1G0053760* (*HSFA2C*) and *SECCE7Rv1G0468390* (*HSFA2D*), were down-regulated in both tissues.

### 3.7 Gene co-expression modules in leaves and roots of ‘White BK-1’ at the early stage of CA

**Figure 6:**
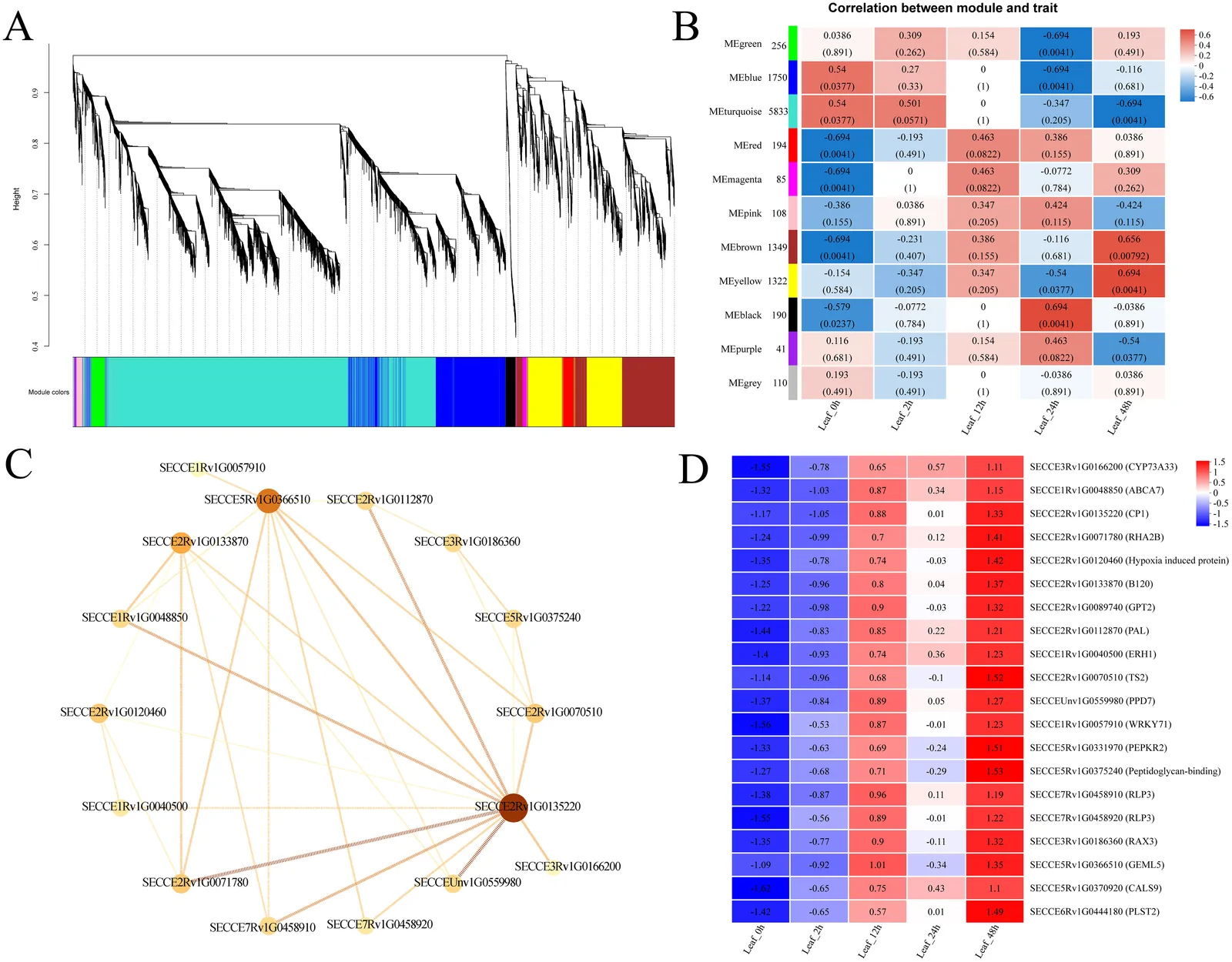
Gene co-expression network analysis of ‘White BK-1’ leaves. **(A)** Hierarchical clustering dendrogram of co-expression modules identified by WGCNA; **(B)** Module-time point associations, with each row representing one module; **(C)** Co-expression networks of the top 20 hub genes with the highest intramodular connectivity in the brown and yellow modules. Edges with TOM-based weights > 0.375 were retained; thicker edges indicate higher weights, and larger nodes indicate greater degree; **(D)** Heatmap showing the expression patterns of the top 20 hub genes.

**Figure 7:**
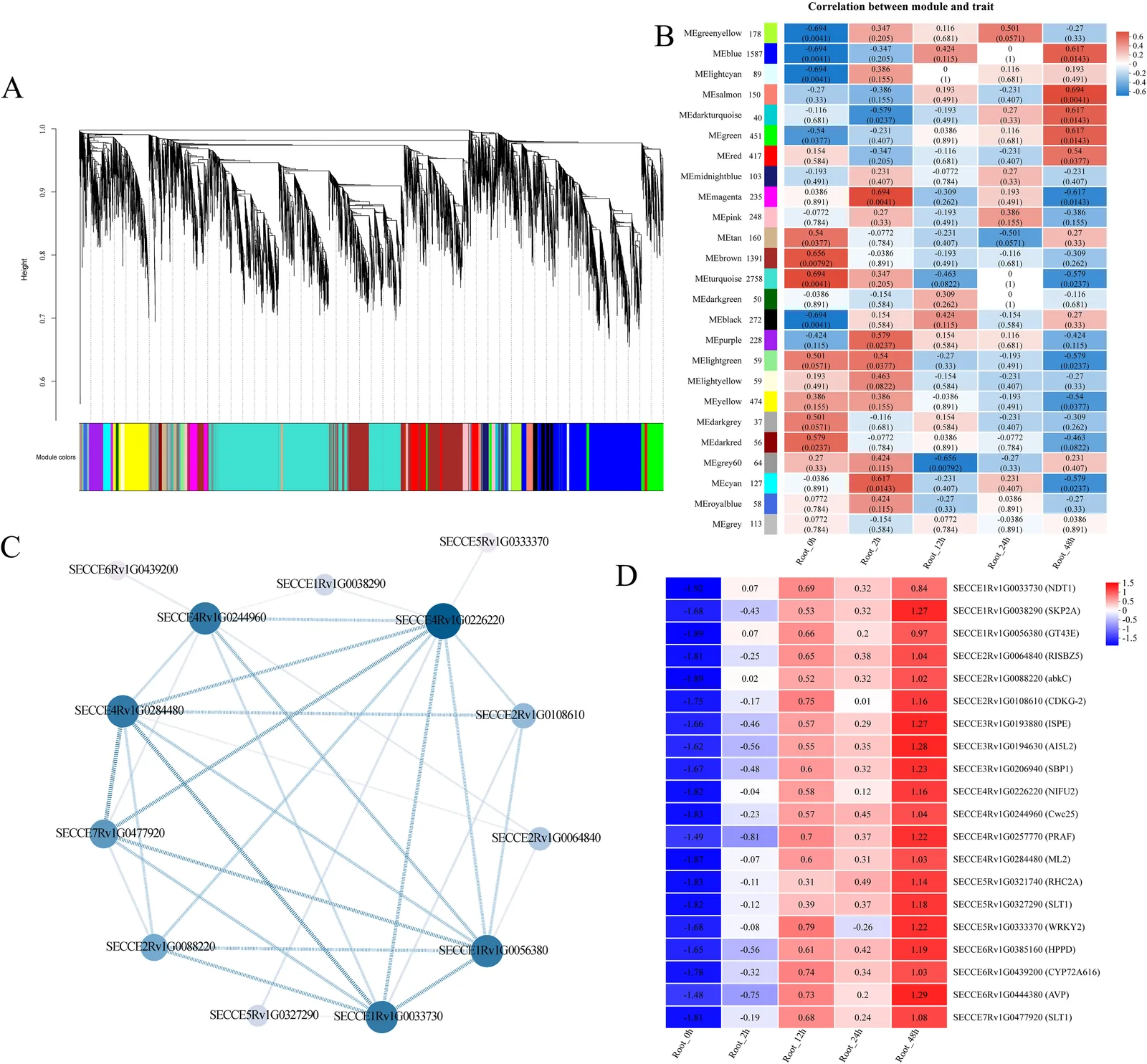
Gene co-expression network analysis of ‘White BK-1’ roots. **(A)** Hierarchical clustering dendrogram of co-expression modules identified by WGCNA; **(B)** Module-time point associations, with each row representing one module; **(C)** Co-expression networks of the top 20 hub genes with the highest intramodular connectivity in the blue and green modules. Edges with TOM-based weights > 0.297 were retained; thicker edges indicate higher weights, and larger nodes indicate greater degree; **(D)** Heatmap showing the expression patterns of the top 20 hub genes.

To investigate the co-expression regulatory networks associated with early CA, WGCNA was performed for leaves and roots using the RNA-Seq data. For network construction, 11,238 genes in leaves and 9,404 in roots were retained. In leaves, these genes were assigned to 11 modules, with the turquoise module containing the largest number of genes (5,833) and the purple module the fewest (41) (**Supplementary Figures 2A, B**; **Figures 6A, B**). In roots, the retained genes were assigned to 25 modules, with the turquoise module containing the most genes (2,758) and the dark-turquoise module the fewest (40) (**Supplementary Figures 2C, D**; **Figures 7A, B**). Module-trait relationship analysis showed that the brown (r = 0.656, *p* = 0.00792) and yellow (r = 0.694, *p* = 0.0041) modules in leaves, as well as the blue (r = 0.617, *p* = 0.0143) and green (r = 0.617, *p* = 0.0143) modules in roots, exhibited significant positive correlations with the 48 h time point, but negative correlations with earlier stages of CA (**Figure 6B**, **7B**), indicating that genes in these modules were likely involved in the later phase of CA. GO enrichment analysis identified 8 CA-responsive genes in the leaf brown and yellow modules, and 15 in the root blue and green modules (**Supplementary Tables 3**, **4**). Notably, two genes, *SECCE5Rv1G0354870* (*COR413PM1*) and *SECCE6Rv1G0415440* (*CS120*), were shared among the four key modules. KEGG enrichment analysis of the leaf brown and yellow modules revealed significant enrichment in Glutathione metabolism, MAPK signaling pathway, and Phenylpropanoid biosynthesis (**Supplementary Figure 3A**). In roots, the blue and green modules were significantly enriched in Plant hormone signal transduction, Linoleic acid metabolism, and other stress-related pathways (**Supplementary Figure 3B**). Further analysis identified 180 DETFs in the key leaf modules and 173 in the key root modules, mainly from the WRKY, ERF, NAC, MYB, MYB-related, bHLH, bZIP, and HSF families. Of these, 57 were shared between leaf and root modules, and most were up-regulated in roots (**Supplementary Figure 4**; **Supplementary Table 5**).

Based on the MM and GS values, we identified 61 candidate DEGs from the leaf key modules and 148 candidate DEGs from the root key modules (**Supplementary Tables 6**, **7**; **Supplementary Figures 5**, **6**). To further identify highly connected genes within these trait-associated modules, co-expression subnetworks were constructed based on TOM among the selected candidate DEGs. Edges with TOM-based weights greater than 0.375 in leaves and 0.297 in roots were retained for network visualization and node-degree analysis. In leaf network, the top 20 genes ranked by connectivity all belonged to the brown module, the five most highly connected genes were *SECCE3Rv1G0166200* (*CYP73A33*), *SECCE1Rv1G0048850* (*ABCA7*), *SECCE2Rv1G0135220* (*CP1*), *SECCE2Rv1G0071780* (*RHA2B*), and *SECCE2Rv1G0120460* (coding a hypoxia-induced protein) (**Figures 6C, D**). Using the same approach in roots, all of the top 20 genes ranked by connectivity were assigned to the blue module. The five highest-ranking genes were *SECCE1Rv1G0033730* (*NDT1*), *SECCE4Rv1G0244960* (*Cwc25*), *SECCE2Rv1G0064840* (*RISBZ5*), *SECCE4Rv1G0226220* (*NIFU2*), and *SECCE4Rv1G0284480* (*ML2*) (**Figures 7C, D**).

### 3.8 Expression dynamics of ICE-CBF-*COR* and hormone-related pathways genes during early CA

**Figure 8:**
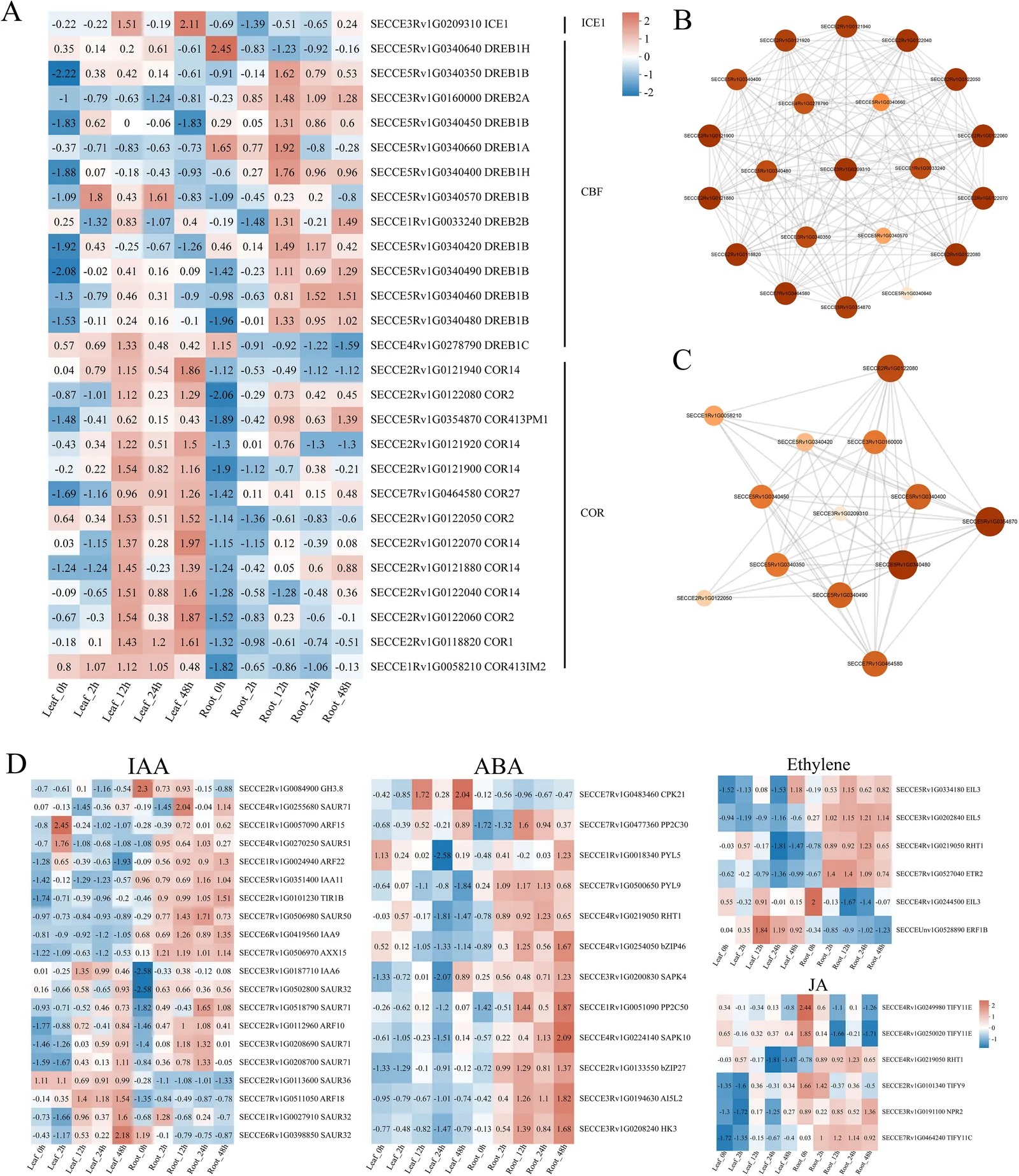
Expression profiles and co-expression networks of ICE-CBF-*COR* pathway genes, and expression profiles of hormone signaling genes in ‘White BK-1’. **(A)** Heatmap of 27 ICE-CBF-*COR* pathway genes in leaves and roots across CA time points; **(B)** Co-expression networks of ICE-CBF-*COR* pathway genes in leaves; **(C)** Co-expression networks of ICE-CBF-*COR* pathway genes in roots; **(D)** Heatmap of IAA-, ABA-, Ethylene-, and JA-signaling genes in leaves and roots across CA time points.

The expression patterns of genes involved the ICE-CBF-*COR* pathway in leaves and roots during the early stage of CA were analyzed, including 1 *ICE1*, 13 *CBF*, and 13 *COR* genes. The heatmap showed that *ICE1* (*SECCE3Rv1G0209310*) expression levels were higher at 12 h and 48 h in leaves, while the overall change was weaker in roots. In addition, the expression levels of several *DREB* genes were significantly increased at 12 h in roots, and some genes still maintained high expression levels at 24 h or 48 h, such as *DREB2A* (*SECCE3Rv1G0160000*) and *DREB1B* (*SECCE5Rv1G0340350*, *SECCE5Rv1G0340450*, *SECCE5Rv1G0340420*, *SECCE5Rv1G0340490*, *SECCE5Rv1G0340460*, *SECCE5Rv1G0340480*). In leaves, most of the *COR* genes, such as *COR14* (*SECCE2Rv1G0121940*, *SECCE2Rv1G0121920*, *SECCE2Rv1G0121900*, *SECCE2Rv1G0122070*, *SECCE2Rv1G0121880*, *SECCE2Rv1G0122040*), *COR2* (*SECCE2Rv1G0122080*, *SECCE2Rv1G0122050*, *SECCE2Rv1G0122060*), *COR27* (*SECCE7Rv1G0464580*), *COR1* (*SECCE2Rv1G0118820*), *COR413PM1* (*SECCE5Rv1G0354870*) were highly expressed at 12 h and 48 h (**Figure 8A**). The co-expression regulatory networks of these 27 genes were established and the regulatory relationships were visualized in leaves and roots, respectively. In leaves, 21 genes had mutual regulatory relationships, and in roots, 13 genes had mutual regulatory relationships. We further examined the distribution and connectivity of ICE-CBF-*COR* related genes within the WGCNA-derived subnetworks. Applying the same TOM-based filtering criterion described above, the leaf subnetwork was more extensive than the root subnetwork. Most ICE-CBF-*COR* related genes in leaves were assigned to the brown module, where *SECCE7Rv1G0464580* (*COR27*) showed the highest connectivity (**Figure 8B**; **Supplementary Table 8**). In roots, these genes were mainly assigned to the blue module, with *SECCE5Rv1G0354870* (*COR413PM1*) showing the highest connectivity (**Figure 8C**; **Supplementary Table 9**). In summary, the ICE-CBF-*COR* pathway showed tissue-specific expression and co-expression patterns during early CA in rye. The leaf subnetwork was more extensive and was dominated by *COR* genes, with *COR27* showing the highest connectivity. In roots, *CBF/DREB-family* genes showed strong induction, and *COR413PM1* was the most highly connected gene in the subnetwork.

DEG, GO, KEGG, and WGCNA analyses indicated that phytohormone signaling plays a key role in the early stages of CA in rye. We therefore examined the expression profiles of genes associated with IAA, ABA, ET, and JA signaling in leaves and roots (**Figure 8D**). The IAA signaling pathway showed distinct expression patterns across tissues and treatment stages. In leaves, *ARF15* (*SECCE1Rv1G0057090*) and *SAUR51* (*SECCE4Rv1G0270250*) were highly expressed at 2 h, whereas *IAA6* (*SECCE3Rv1G0187710*), *SAUR71* (*SECCE3Rv1G0208690*, *SECCE3Rv1G0208700*), *SAUR36* (*SECCE2Rv1G0113600*), *SAUR32* (*SECCE1Rv1G0027910*, *SECCE6Rv1G0398850*), and *ARF18* (*SECCE7Rv1G0511050*) showed higher expression at 12 h, 24 h, and 48 h. In roots, *TIR1B* (*SECCE2Rv1G0101230*), *IAA9* (*SECCE6Rv1G0419560*), *SAUR50* (*SECCE7Rv1G0506980*), *AXX15* (*SECCE7Rv1G0506970*), *IAA11* (*SECCE5Rv1G0351400*), and *ARF22* (*SECCE1Rv1G0024940*) were preferentially expressed. ABA signaling was more prominent in roots and showed a rapid response to cold treatment. *PYL9* (*SECCE7Rv1G0500650*), *RHT1* (*SECCE4Rv1G0219050*), *bZIP46* (*SECCE4Rv1G0254050*), *SAPK4* (*SECCE3Rv1G0224140*), *SAPK10* (*SECCE4Rv1G0224140*), *bZIP27* (*SECCE2Rv1G0133550*), *AI5L2* (*SECCE3Rv1G0194630*), and *HK3* (*SECCE3Rv1G0208240*) showed increased transcript abundance from 2 h onward. *PP2C30* (*SECCE7Rv1G0477360*) and *PP2C50* (*SECCE1Rv1G0051090*) were highly expressed at 12 h. ET and JA signaling also exhibited clear tissue specificity. In the ET signaling pathway, *ERF1B* (*SECCEUnv1G0528890*) reached its highest expression level in leaves at 12 h and then gradually declined. *EIL3* (*SECCE5Rv1G0334180*), *EIL5* (*SECCE3Rv1G0202840*), *RHT1* (*SECCE4Rv1G0219050*), and *ETR2* (*SECCE7Rv1G0527040*) maintained high expression in roots during cold treatment. In the JA signaling pathway, *RHT1* (*SECCE4Rv1G0219050*), *TIFY11C* (*SECCE7Rv1G0464240*), and *NPR2* (*SECCE3Rv1G0191100*) were mainly expressed in roots. These results indicated that hormone signaling during early CA in rye was characterized by pronounced tissue specificity. IAA signaling displayed divergent expression patterns between leaves and roots, whereas ABA, ET, and JA responses were more pronounced in roots. Notably, *RHT1* (*SECCE4Rv1G0219050*), which encodes a DELLA repressor, was identified in multiple pathways and was preferentially expressed in roots, suggesting that growth regulation mediated by DELLA proteins may contribute to hormone crosstalk and cold adaptation in rye roots.

### 3.9 Verification of genes by qRT-PCR

**Figure 9:**
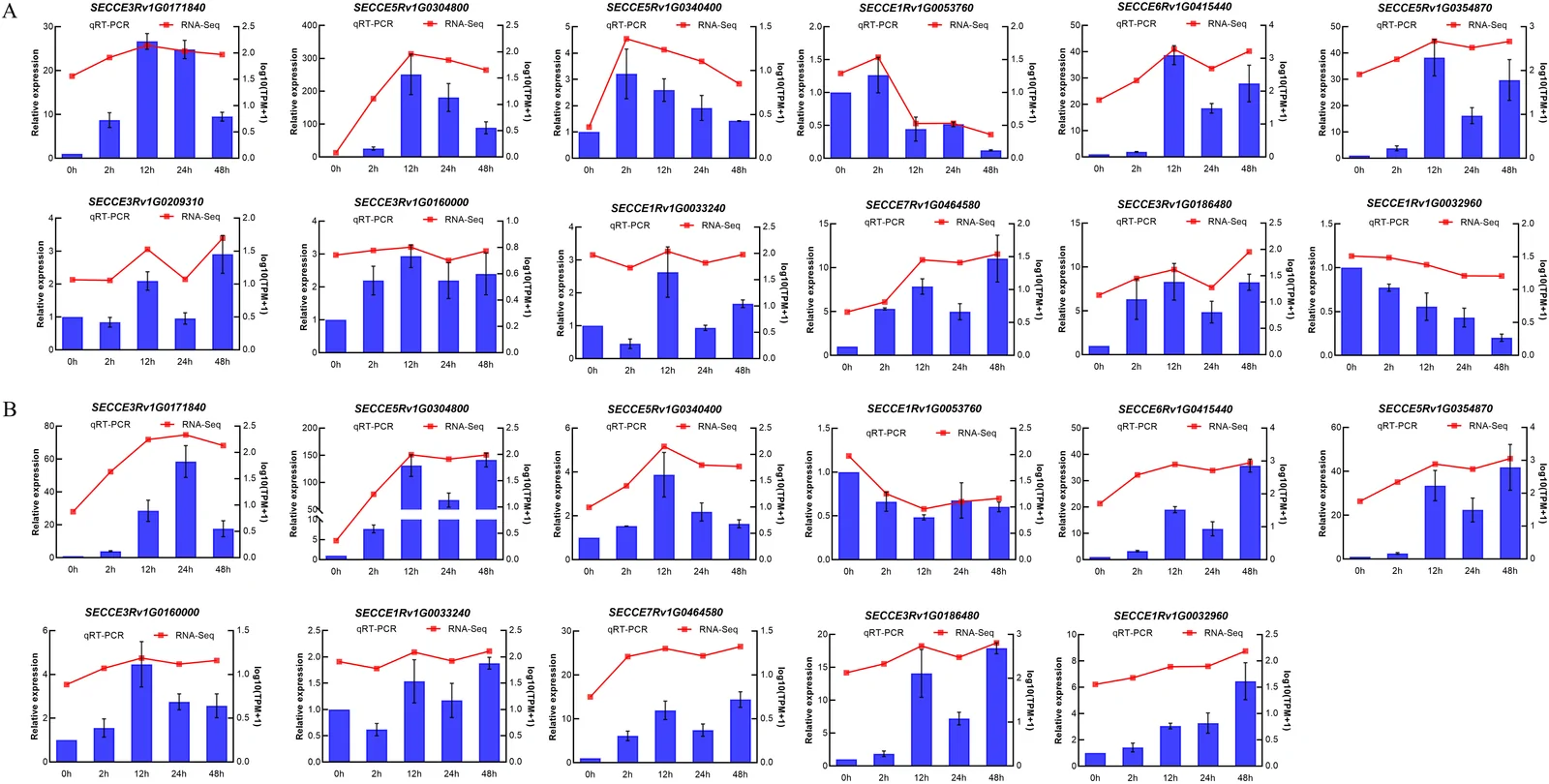
Expression patterns of selected candidate genes in leaves **(A)** and roots **(B)** during the 0–48 h CA period. In each panel, the left y-axis indicates the relative expression levels determined by qRT-PCR, and the right y-axis represents the transcript abundance from RNA-Seq data quantified as log_10_ (TPM + 1). Error bars represent the standard deviation (SD) of technical replicates. The *ScActin* was used as an internal control for normalization.

To validate the RNA-Seq results, we selected 12 candidate genes representing different analytical dimensions of this study, including TFs up-regulated across both tissues, namely *SECCE5Rv1G0340400* (*DREB1H*), *SECCE5Rv1G0304800* (*CPRF2*), and *SECCE3Rv1G0171840* (*CDF2)*; a TF down-regulated in both tissues, *SECCE1Rv1G0053760* (*HSFA2C*); hub genes shared across the key WGCNA modules in leaves and roots, *SECCE5Rv1G0354870* (*COR413PM1*) and *SECCE6Rv1G0415440* (*CS120*); components of the ICE-CBF-*COR* pathway, *SECCE3Rv1G0209310* (*ICE1*), *SECCE3Rv1G0160000* (*DREB2A*), *SECCE1Rv1G0033240* (*DREB2B*), and *SECCE7Rv1G0464580* (*COR27*); a tissue-specific TF, *SECCE1Rv1G0032960* (*MYBS3*); and a metabolic gene encoding glutamate synthase 1, *SECCE3Rv1G0186480* (*GLT1*). QRT-PCR analysis showed that the expression patterns of all 12 genes were highly consistent with the RNA-Seq data across tissues and time points (**Figure 9**; **Supplementary Figure 7**).

## 4 Discussion

CA is essential for winter survival in temperate cereals because it enables plants to acquire freezing tolerance before the onset of severe winter stress (Thomashow, 1999; Liu et al., 2019). A central feature of this process is the adjustment of growth with the buildup of stress protection (Mahajan and Tuteja, 2005; Janská et al., 2010). In this study, CA enhanced freezing tolerance in ‘White BK-1’ and was accompanied by reductions in RHGR and RBGR (**Figure 1**). The trade-off between plant growth and stress adaptation is that plants build their own protective capacity while reducing their continuous accumulation of biomass. Studies have shown that, under abiotic stress, a decrease in RHGR and RBGR helps suppress excessive plant growth while allowing resources to be reallocated to the regulation of the photosynthetic system and antioxidant defense processes (Vaitkevičiūtė et al., 2022; Zhou et al., 2023; Dusfour-Castan et al., 2026). Characterizing tissue-specific transcriptional responses of ‘White BK-1’ during early CA may help identify early cold-responsive genes and candidate upstream regulators.

### 4.1 The differences in tissue and temporal responses of ‘White BK-1’ during the early stages of CA

Comparison of DEG numbers in leaves and roots showed that the transcriptomic response of ‘White BK-1’ changed during the early stages of CA (**Figure 2**). The enrichment results also pointed to clear differences between the two tissues. In leaves, the early representation of chloroplast-related terms and the Photosynthesis pathway suggest that photosynthesis-related genes responded rapidly to cold exposure (**Figures 3A**, **4A**). This is potentially important because photosynthesis provides energy and carbon skeletons for a range of metabolic and acclimatory processes (Lai et al., 2025). At the same time, low temperature can constrain carbon assimilation more strongly than light capture, creating a potential imbalance between absorbed light energy and its metabolic utilization. Such an imbalance has been associated with excess excitation energy and oxidative stress under cold conditions (Giró et al., 2011; Seydel et al., 2022). Against this background, the later enrichment of glutathione metabolism, phenylpropanoid- and anthocyanin-related pathways, and lipid-related pathways may reflect a transcriptional adjustment involving redox regulation, secondary metabolism, and membrane-associated processes. This interpretation is in agreement with our previous physiological observations, in which ‘White BK-1’ maintained relatively high net photosynthetic rates and antioxidant enzyme activities under low-temperature stress (Yang et al., 2024b).

The root response differed from that observed in leaves. Early changes were mainly associated with transcriptional regulation, whereas water-deficit-, ABA-, and metabolism-related categories became more prominent at later time points (**Figure 3B**). The significant enrichment of functional terms related to water deprivation and ABA response is consistent with the physiological status of roots under low temperature conditions. Low temperature can reduce membrane fluidity and water transport capacity in roots, thereby inhibiting root water uptake and inducing drought- or dehydration-like signals. Previous studies have also shown that plant responses to low temperature and drought are not completely independent, but share some components involved in stress perception, signal transduction, and transcriptional regulation (Jin et al., 2017b; Wang et al., 2025a). Nevertheless, further physiological and functional validation is required to determine whether these transcriptional patterns are associated with dehydration-like responses in roots. The enrichment of nitrogen- and amino-acid-related pathways suggests transcriptional changes associated with carbon-nitrogen balance during the early stages of CA; however, whether these changes lead to physiological or metabolic adjustments requires further validation. At 48 h, pathway enrichment analysis suggested potential transcriptional reprogramming associated with cellular homeostasis and metabolic regulation in roots, although the functional significance of this reprogramming remains to be validated.

### 4.2 Tissue-specific transcriptional pattern during the early stages of CA

TFs regulate the expression of stress-responsive genes by binding to cis-acting elements in the promoters of target genes. Previous studies have shown that several TF families are involved in plant responses to abiotic stress, including the DREB/CBF subfamily of the AP2/ERF family, the ABF/AREB subfamily of the bZIP family, and members of the NAC, MYB, bHLH, WRKY, and C2H2 zinc finger families (Meraj et al., 2020; Kidokoro et al., 2022; Zhang et al., 2024). In ‘White BK-1’, DETFs showed distinct temporal patterns between leaves and roots (**Figures 5A–C**). Notably, *CPRF2* and *CDF2* were consistently upregulated in both leaves and roots during early CA, suggesting their involvement in the rye CA response. *CPRF2* is a G-box-binding bZIP TF implicated in light/UV-responsive phenylpropanoid metabolism and light-induced transcription, whereas *CDF2* has been associated with CA in Arabidopsis. However, their functions and regulatory mechanisms in rye remain unclear (Kircher et al., 1999; Wellmer et al., 1999; Fornara et al., 2009, Fornara et al., 2015). ERF members represented the largest group at each time point in both leaves and roots, suggesting a broad ERF related transcriptional response to low temperature in rye. DREB/CBF members of the AP2/ERF family are known as important regulators of plant responses to cold stress (Park et al., 2015; Meraj et al., 2020). Therefore, we further examined the classical ICE-CBF-*COR* pathway (**Figures 8A–C**). The predominance of AP2/ERF factors and the induction of multiple *DREB1B-like* genes in roots suggest the involvement of CBF-related regulation in the root response. The induction of *DREB2A* may additionally reflect dehydration- or osmotic-stress signaling, consistent with the enrichment of water-deprivation- and ABA-responsive terms in roots. Unlike in leaves, *SECCE3Rv1G0209310* (*ICE1*) was not significantly induced in roots, consistent with the weaker cold-induced expression of *TaICE1* in wheat roots than in leaves (Szalai et al., 2025). This pattern suggests that root *DREB/CBF* induction cannot be fully explained by ICE1 transcriptional upregulation alone. This does not exclude a role for ICE1, as its activity is extensively regulated by post-translational modifications, including ubiquitination, SUMOylation, and phosphorylation, which affect protein stability and transcriptional activity under cold stress (Ding et al., 2024). Moreover, *CBF* expression can be modulated by Ca^2+^-related signaling, CAMTA, ABA/SnRK2 signaling, MAPK cascades, and BES1/BZR1 proteins (Li et al., 2017; Shi et al., 2018; Yuan et al., 2018). Together, these observations suggest that root *DREB/CBF* induction may integrate multiple cold-signaling pathways rather than depend solely on transcriptional activation of *ICE1*. Protein-level analyses will be required to determine whether ICE1 abundance, modification status, or activity changes during CA in rye roots.

Furthermore, the ICE-CBF-*COR*-related co-expression networks indicated that *SECCE7Rv1G0464580* (*COR27*) showed the highest connectivity in leaves (**Supplementary Table 8**). Previous studies have associated *COR27* with plant cold responses and circadian-related regulation, suggesting that the leaf network may be associated with the coordination between low-temperature response and rhythmic regulation (Wang et al., 2017; Zhu et al., 2020). In roots, *SECCE5Rv1G0354870* (*COR413PM1*) showed the highest connectivity (**Supplementary Table 9**). COR413PM proteins have been proposed to contribute to plasma membrane stability under low-temperature conditions, and overexpression of *SikCOR413PM1* in tobacco resulted in enhanced tolerance to cold and drought stress (Su et al., 2018; Zhou et al., 2018; Guo et al., 2019). Furthermore, *AtCOR413PM1* has been identified as a gene involved in ABA-mediated regulation, and mutation of *AtCOR413PM1* downregulates the expression of several ABA-responsive genes (Hu et al., 2021). In rye, variation in *COR413PM* genes has also been associated with winter field survival (Båga et al., 2022). The central position of *COR413PM1* in the rye root network may be related to membrane-associated cold protection, and the biological function of this gene needs to be verified in the future.

### 4.3 Tissue-specific hormone-related transcriptional responses during the early stages of CA

Phytohormones are small molecule chemicals, which have been proved to play key roles in plant cold stress response (Shi et al., 2015; Verma et al., 2016). Genes associated with several major phytohormone signaling pathways displayed different expression patterns in rye leaves and roots during early CA stage (**Figure 8D**). ABA is a central hormonal signal linking temperature stress with osmotic adjustment and water balance, and its downstream signaling is known to induce genes associated with dehydration protection, osmotic regulation, and stress-responsive transcriptional control. In the ABA pathway, stress responses involve PYR/PYL receptors, PP2C phosphatases, SnRK2/SAPK kinases, and BZIP/ABF (Xue-Xuan et al., 2010; Fujita et al., 2013; Yu et al., 2020). In this study, multiple ABA signaling-related genes were strongly induced in roots after cold treatment, indicating that ABA-mediated signaling forms an important part of rye root cold-response network and may contribute to the activation of downstream protective mechanisms during early CA. Future studies should clarify whether this pathway is required for rye cold tolerance and identify the key downstream regulators and target genes involved. Particular attention should be paid to *SECCE4Rv1G0219050* (*RHT1*), which was annotated as encoding a DELLA protein. DELLA proteins are well known as central repressors in GA signaling and have also been implicated in the coordination of growth restraint and stress adaptation through interaction with other hormone pathways (Shi et al., 2015; Lantzouni et al., 2020). In wheat, *Rht1* and *Rht2* have been widely used in breeding programs and have made important contributions to the improvement of wheat grain yield (Mohan et al., 2021). In particular, overexpression of *RHT1* significantly reduces plant height, while increasing tiller number and tiller angle (Xu et al., 2023). Recent evidence further indicates that *RHT* alleles can also influence wheat seedling root architecture and root-shoot balance (Wang et al., 2025b). The induction of *RHT1* in rye roots raises a possibility about GA-related transcriptional involvement, together with hormone crosstalk involving ABA, ET, and JA pathways, may contribute to root-specific adjustment during CA. At the same time, DELLA function is strongly influenced by post-transcriptional regulation and protein stability, so the biological significance of *SECCE4Rv1G0219050* (*RHT1*) induction in rye roots will require further validation.

We acknowledge that this study has limitations. Because the analysis was based on a single genotype, the findings may not fully reflect variation across genotypes. Moreover, the co-expression relationships identified here are correlational and should not be interpreted as evidence of direct regulatory interactions. The candidate genes and network relationships identified in this study therefore require functional validation in future experiments.

## 5 Conclusion

In conclusion, our study showed that CA in rye was accompanied by increased freezing tolerance and reduced plant growth, indicating a potential association between growth restraint and freezing tolerance. Transcriptome analysis revealed tissue-specific transcriptional responses in rye roots and leaves during the first 48 h of CA. In roots, the response involved genes related to transcriptional regulation, hormone-associated processes, and metabolism. In leaves, enriched functions were mainly related to photosynthesis at 2 h, whereas genes associated with protective metabolite biosynthesis became more prominent by 48 h. In the ICE-CBF-*COR* signaling pathway, *SECCE7Rv1G0464580* (*COR27*) and *SECCE5Rv1G0354870* (*COR413PM1*) were identified as the most highly connected genes in the leaf and root subnetworks, respectively. In addition, *SECCE5Rv1G0340400* (*DREB1H*), *SECCE5Rv1G0304800* (*CPRF2*), and *SECCE3Rv1G0171840* (*CDF2*) were upregulated in both tissues, suggesting that they may represent candidate shared regulators during early CA. Hormone-related genes, particularly those associated with ABA signaling, were upregulated in roots, suggesting that ABA-related signaling may be involved in rye root response to CA. Notably, *SECCE4Rv1G0219050* (*RHT1*), a putative DELLA-encoding gene, was highly expressed in roots and was associated with ABA, ET, and JA signaling. These expression patterns suggest that *RHT1* is a candidate gene for further investigation during CA.

Taken together, these findings provide insight into the distinct transcriptional responses of roots and leaves during early CA in rye and identify candidate genes and co-expression modules potentially relevant to freezing tolerance in cereals.

## Data Availability

The data presented in the study are deposited in the National Center for Biotechnology Information (NCBI) Sequence Read Archive (SRA) database, accession number PRJNA1494119.
