# Supplementary material for: Time-course transcriptome and WGCNA reveal tissue-specific genes in ‘White BK-1’ rye during early cold acclimation stage
Source: Front Plant Sci. 2026 Sep 7;17:1936346. doi: 10.3389/fpls.2026.1936346 (PMC13590303; doi:10.3389/fpls.2026.1936346)
Supplement: Supplementary file 1 [file DataSheet1.zip › Supplementary Figures.docx]

Figure S1 KEGG pathway enrichment analysis of down-regulated genes in leaves (A) and roots (B) during CA.

Figure S2 Scale Free Topology Fit index and Mean Connectivity curves for soft-threshold power selection in WGCNA of White BK-1 leaves (A, B) and roots (C, D).

Figure S3 KEGG enrichment analysis of genes within key WGCNA modules in leaves and roots. (A) Genes in the leaf yellow and brown modules; (B) Genes in the root blue and green modules.

Figure S4 Identification and expression profiling of common TFs associated with key WGCNA modules in leaves and roots. (A) Venn diagram showing common TFs among DETFs, TFs from the yellow and brown modules in leaves, and TFs from the blue and green modules in roots; (B) Heatmap showing the expression patterns of the common TFs.

Figure S5 Identification of candidate hub genes among DEGs in the brown and yellow leaf modules at 0 h and 48 h based on MM-GS analysis, with scatterplots shown for the brown module at 0 h (A) and 48 h (B), and the yellow module at 0 h (C) and 48 h (D); Black dashed lines indicate the thresholds used to identify candidate hub genes.

Figure S6 Identification of candidate hub genes among DEGs in the green and blue root modules at 0 h and 48 h based on MM-GS analysis, with scatterplots shown for the green module at 0 h (A) and 48 h (B), and the blue module at 0 h (C) and 48 h (D); Black dashed lines indicate the thresholds used to identify candidate hub genes.

Figure S7 Pearson correlation analysis between RNA-seq and qRT-PCR expression changes in leaves (A) and roots (B). The x-axis shows qRT-PCR-derived log_2_ fold changes (FC) calculated using the 2^−ΔΔCt^ method, and the y-axis shows RNA-seq-derived log_2_ FC; Pearson’s r and significance level are shown in each panel.
